# One-Pot Synthesis of Lactose Derivatives from Whey Permeate

**DOI:** 10.3390/foods9060784

**Published:** 2020-06-13

**Authors:** Maryam Enteshari, Sergio I. Martínez-Monteagudo

**Affiliations:** Dairy and Food Science Department, South Dakota State University, Alfred Dairy Science Hall, Brookings, SD 57007, USA; Maryam.Enteshari@sdstate.edu

**Keywords:** one-pot synthesis, catalysis, lactulose, lactobionic acid

## Abstract

The simultaneous production of lactulose (LAU), lactobionic acid (LBA), and organic acids from sweet and acid whey permeate (SWP and AWP) via catalytic synthesis (5% Ru/C) was studied in a continuous stirred-tank reactor. At selected conditions (60 °C, 60 bar, and 600 rpm), a maximum conversion of lactose (37 and 34%) was obtained after 90 min for SWP and AWP, respectively. The highest yield calculated with respect to the initial concentration of lactose for LAU was 22.98 ± 0.81 and 15.29 ± 0.81% after only 30 min for SWP, and AWP, respectively. For LBA, a maximum yield was found in SWP (5.23%) after 210 min, while about 2.2% was found in AWP. Six major organic acids (gluconic, pyruvic, lactic, formic, acetic, and citric acid) were quantified during the one-pot synthesis of lactose.

## 1. Introduction

Whey is the main byproduct obtained from the manufacture of cheese, yogurt, and milk protein concentrates [1]. It is the yellowish liquid separated from curds, and it is mainly made of water (~94%), lactose (~5%), proteins (~1%), minerals (~1%), and milk fat (~0.5%) [2]. As a byproduct, whey is concentrated and further fractionated to produce a wide array of products and ingredients with food and pharmaceutical applications. Examples of ingredients derived from whey are milk serum protein concentrate, whey powder, whey protein concentrate, whey protein hydrolysate, and whey protein isolate. Manufacturing details on the production of milk protein ingredients can be found elsewhere [3,4].

Over the last few years, the number of food formulations containing milk proteins has considerably increased due to the functional and nutritional properties of milk proteins [5]. Nowadays, products such as sport drinks, desserts, beverages, snacks, dietary, and weight management supplements are formulated with milk proteins. Overall, the manufacture of concentrates of milk proteins involves numerous unit operations, such as thermal treatment, selective fractionation, concentration, and drying. Selective fractionation by means of membrane technology is the key processing step in which the protein fraction is sequentially separated from the whey stream. The separation is performed by multiple filtration stages, where the proteins are concentrated in the retentate, while lactose and minerals are concentrated in permeate stream. The byproduct derived from the production of milk proteins is known as whey permeate, and it contains a considerable amount of lactose (75–80% on dry basis).

**Figure 1:**
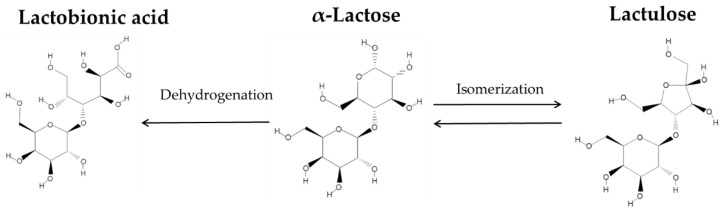
Reaction pathway for the formation of lactulose and lactobionic acid via isomerization and dehydrogenation, respectively.

Lactose (LA) presents technological challenges, such as poor solubility and low sweetness index, as well as malabsorption by a certain population, which has limited its further utilization [6]. Consequently, there is a considerable industrial interest to further utilize LA as a feedstock for the production of lactose-based ingredients [7,8]. As a feedstock, lactose has the potential to undergo different reactions leading to the formation of value-added compounds. Figure 1 illustrates the simultaneous formation of lactulose (LAU) and lactobionic acid (LBA), two of the most versatile lactose-derived ingredients, with applications in food, dairy, and pharmaceutical formulations.

Lactulose (4-*O*-β-d-galactopyranosyl-d-fructofuranose), an isomer of LA, is a disaccharide made of galactose and fructose. It is used in the treatment of hepatic encephalopathy and chronic constipation [9]. Industrially, LAU is synthesized by either homogenous catalysis under alkaline conditions, or by enzymatic synthesis using glycosyltransferases and glycosidases [10]. Reaction schemes and conditions for LAU synthesis can be found elsewhere [11]. On the other hand, LBA (4-*O*-β-galactopyranosyl-d-gluconic acid) is an aldonic acid or sugar-acid made up of galactose linked to a gluconic acid [12]. The LBA molecule has various functionalities, such as antioxidant, chelating, and humectant, arising from the high number of hydroxyl groups. The partial oxidation of LA results in the formation of LBA, where the aldehyde group is converted into its corresponding carbonyl group via selective oxidation [13]. Most of the LBA commercialized in the world is produced via 1) biocatalytic conversion using dehydrogenase or oxidoreductase, or 2) heterogeneous catalysis using Pd, Pt, or Ru as insoluble catalysts. The manufacturing methods of LBA can be found elsewhere [12].

One common strategy employed in the industrial production of lactose-derived ingredients is the use of purified lactose (monohydrate or pharmaceutical grade) as a feedstock, which minimizes the formation of secondary products. Fundamentally, such a strategy aims to produce a single compound via multi-step flow, involving the formation, separation, and isolation of secondary and primary products. Alternatively, the conversion of lactose through one-pot synthesis represents an innovative utilization of lactose. One-pot conversion involves the synthesis of a mixture of compounds that can be used with minimal separation in the final formulation. This has been exemplified by our group, where a sweetening syrup from aqueous lactose was produced via one-pot synthesis (enzymatic hydrolysis followed by catalytic isomerization) [14,15]. Similarly, Zaccheria [16] produced a mixture of sorbitol and dulcitol from lactose via one-pot catalytic conversion. Gallezot [17] conceptualized and exemplified the production of a pool of molecules from biomass via one-pot catalytic synthesis. Under the one-pot approach, the high concentration of lactose present in whey permeate can be used as an inexpensive feedstock for a variety of chemical modifications, such as hydrogenation, isomerization, and oxidation.

During the catalytic oxidation of lactose, the first products formed are LBA and LAU, followed by 2-keto-lactobionic acid as a secondary product [18]. Preliminary results showed that increasing the oxygen pressure improved the yield of LAU during the oxidation of lactose over Ru/C [19]. It should be highlighted that the production of LAU and LBA was evaluated using lactose monohydrate as a feedstock. Nevertheless, these results present an opportunity for the simultaneous production of LAU and LBA directly from whey permeate. This work aimed at producing LAU and LBA via a one-pot catalytic oxidation from sweet and acid whey permeate.

## 2. Materials and Methods

### 2.1. Materials

Firstly, α-d-Lactose monohydrate (98%, Acros Organics, Fair Lawn, NJ, USA), lactulose (99%, Alfa Aesar, Haverhill, MA, USA), lactobionic acid (97%, Sigma-Aldrich, St. Louis, MO, USA), D-(+)-glucose (99%, Sigma-Aldrich), D-(+)-galactose (99%, Sigma-Aldrich), D-gluconic acid sodium salt (99%, Sigma-Aldrich), pyruvic acid (98%, Sigma-Aldrich), L-(+)-lactic acid (85%, Sigma-Aldrich), formic acid (99%, Fisher Scientific, Waltham, MA, USA), citric acid (99%, Sigma-Aldrich), and orotic acid (98%, Sigma-Aldrich) were purchased from commercial suppliers. Reduced ruthenium supported on activated carbon (5% Ru/C, Alfa Aesar) was purchased from commercial suppliers, and it was used without further preparation. Sweet whey permeate (SWP) was obtained from a regional cheese factory (Valley Queen Co., Milbank, SD, USA), while the acid whey permeate (AWP) was obtained from a Greek yogurt plant (Chobani Co., Twin Falls, ID, USA).

### 2.2. Preparation of Samples

A 10% wt./wt. solution of lactose (LAS) was prepared by dissolving D-lactose monohydrate in distilled water. Samples of SWP and AWP were analyzed for pH, total protein, fat content, total solids, and total non-volatile solids. The pH was measured in 10 mL of the sample using a triode epoxy electrode (Orion Versa Star Pro, Thermo Fisher Scientific). The protein content was determined by the Kjeldahl method, while the fat content was measured using the Mojonnier fat extraction, following the guidelines of the Association of Official Analytical Chemists (AOAC) methods 989.05 and 991.20, respectively [20]. Total solids (TS) and total non-volatile solids (TNVS) were determined using the AOAC method 990.20 [20]. Table 1 shows the compositional characteristics of SWP and AWP.

### 2.3. Experimental Treatments

A set of experiments was first conducted to evaluate the influence of pressure (15, 40, 60, and 80 bar) and temperature (50, 60, 70, and 80 °C) on the conversion rate of lactose in a 10% wt./wt. solution of lactose. Initial rates (r_o_) of lactose conversion were calculated using Equation (1):

(1) $${r_{o}=\frac{d\left[La\right]}{dt}|}_{t=0}$$

At selected conditions, the second set of experiments was conducted to evaluate the formation of LAU and LBA directly from sweet whey permeate (SWP), and acid whey permeate (AWP).

### 2.4. One-Pot Synthesis

**Figure 2:**
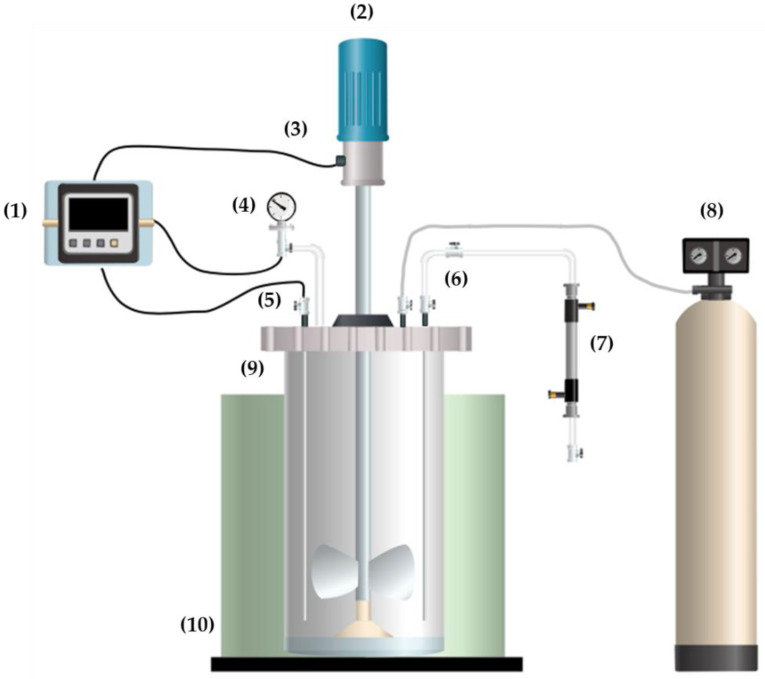
Schematic of the continuous stirred-tank reactor used for the one-pot conversion of lactose permeate: (1) data logger, (2) stirrer, (3) tachometer, (4) pressure gauge, (5) thermocouple, (6) sampling port, (7) cooling system, (8) nitrogen cylinder, (9) stainless-steel reactor, (10) electrical heater.

The one-pot conversion of LA was carried out in a continuous stirred-tank reactor (BR-300, Berghof Products & Instruments, Berghof, Germany). Figure 2 schematically represents the one-pot conversion of lactose directly from whey permeate. Features and characteristics of the reactor, as well as the working conditions, have been explained thoroughly in previous work [21]. Briefly, the reactor vessel was loaded with 400 mL of either LAS, SWP, or AWP, containing 0.5 g/L of Ru/C catalyst. Then, the vessel was lidded, clamped, and heated up to the target temperature. During heating, the vessel was pressurized with compressed air as an oxidizing agent (99.99% purity, Praxair, Sioux Falls, SD, USA). The initial time of the catalytic reaction was considered when the solution reached the target temperature and pressure. Once the working conditions were obtained, the reaction was monitored over 210 min, withdrawing samples (~15 mL) every 30 min and stored at −20 °C until further analysis. Samples were withdrawn from the sampling port ((6) in Figure 2), consisting of a set of needle valves that allowed the subtraction of samples without losing pressure inside the reactor.

### 2.5. Quantification of Reaction Products

The reaction products derived were quantified by liquid chromatography-mass spectrophotometry (LC-MS). Firstly, samples withdrawn from the reactor were filtered through a 20-25 μm filter (NO 541 Hardened Ashless, and 110 mm diameter, WhatmanTM Co., Marlborough, MA, USA) to remove the spent catalyst. Then, the filtered solution was diluted 10-fold with HPLC grade water, followed by centrifugation using ultracentrifugation filters (Amicon^®^ filters, Merc Millipore, Billerica, MA, USA) at 9000 rpm for 10 min (accuSpin Micro 17R, Fisher Scientific, Waltham, MA, USA). After centrifugation, the precipitate was discarded, while the supernatant was used for analysis. An aliquot of 10 µL from the supernatant was injected into a Shimadzu LC system (LC-20AD, Shimadzu Corp, Kyoto, Japan), combined with a Qtrap 5500 triple quadrupole mass spectrometer (AB Sciex, Foster City, CA, USA). Analytes were eluded by means of an HPX-87C column (250x4.0 mm, Bio-Rad Aminex, Hercules, CA, USA) operated at 80 °C with a mobile phase consisted of acetonitrile: water (20:80 v/v), at a flow rate of 0.2 mL/min. Mass spectrophotometer analysis was operated in negative mode at a temperature of 500 °C, a curtain gas of 30 psi, an ion source gas of 15 psi for nebulizer (GS1), and heater (GS2). Samples were quantified according to HPLC-grade analytical standards.

### 2.6. Quantification of Organic Acids

The organic acids formed during the catalytic oxidation of LA were analyzed by HPLC, according to the methodology reported by Zeppa et al. [22], with some modifications. An HPLC instrument (Beckman Coulter, Inc., Fullerton, CA, USA) equipped with a solvent delivery module (System Gold^®^ 125), a multichannel wavelength scanning detector (190–600 nm, System Gold 168 detector), and a refractive index detector (RI-2031, Jasco Corporation, Hachioji, Japan) were used for the analysis. The separation of organic acids was performed using an ion exclusion column (ROA-Organic Acid H+ 8%, Phenomenex, Torrance, CA, USA) heated at 60 °C. The mobile phase was a sulfuric acid solution (0.013 N), at a flow rate of 0.5 mL min^−1^.

### 2.7. Data Analysis

Lactose conversion was expressed as a percentage of converted LA into its derivatives, according to Equation (2).

(2) $$C_{LA}=\frac{\left[LA_{o}\right]-\left[LA_{t}\right]}{\left[LA_{o}\right]}\times 100$$

where $C_{LA}$—conversion of lactose (%), $\left[LA_{o}\right]$—initial concentration of lactose (mM), and $\left[LA_{t}\right]$—concentration of lactose at a given reaction time. The formation of LAU and LBA was determined as the product yield ($Y_{i}$) according to Equation (3).

(3) $$Y_{i}=\frac{Concentration\;of\;target\;product}{Initial\;concentration\;of\;lactose}\times 100$$

Experimental runs were conducted in duplicate, and all the figures were made with SigmaPlot software V11 for Windows (Systat Software, Inc., Chicago, IL, USA).

## 3. Results and Discussion

### 3.1. Reaction Conditions

**Figure 3:**
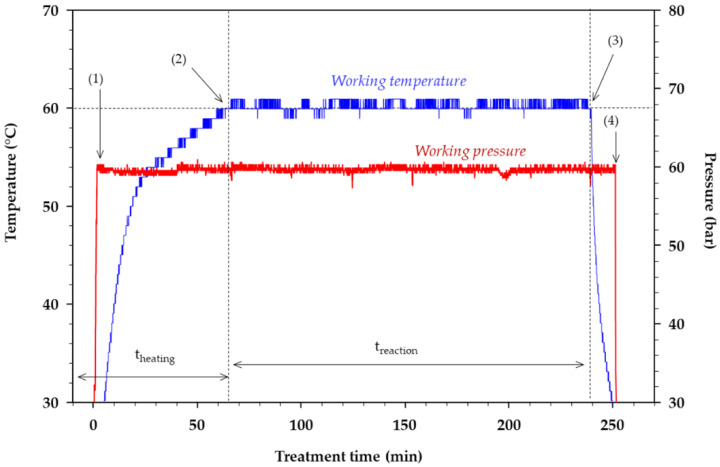
Representative temperature-pressure history during the one-pot conversion of lactose. Reaction temperature was 60 °C and pressure was 60 bar. t_heating_—heating time (min), and t_reaction_—reaction time (min). Arrows (1–4) represent the start of pressurization, the beginning of the reaction time, the end of the reaction time, and the depressurization, respectively.

Figure 3 shows a graphical representation of the temperature-pressure history during the conversion of LA. Each experimental treatment started with the heading time, which includes loading, pressurization (arrow (1)), and heating of the vessel. Start of the reaction time (arrow (2)) was considered when both the target temperature and pressure were achieved. Afterward, samples (~15 mL) were withdrawn for analysis at interval times of 30, 90, 150, and 210 min. The end of the reaction time (arrow (3)) marked the beginning of the cooling and depressurization (arrow (4)). Similar characterization of the temperature-pressure history was reported elsewhere [21]. A detailed description of the main variables involved during the one-pot conversion allows one to meaningfully interpret the formation of reaction products, and facilitates comparison with the literature.

### 3.2. Initial Rates

**Figure 4:**
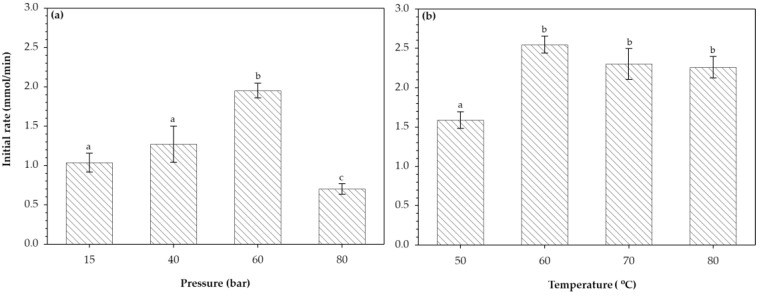
Initial rate of lactose conversion: (**a**) pressure effect (temperature = 60 °C) and (**b**) temperature effect (pressure = 60 bar). Mean ± standard deviation within treatments with different letters are (**a**–**c**) significantly different (*p* < 0.05) according to Tukey’s test.

The initial rate of LA conversion is shown in Figure 4. Pressure influence on the initial rates was evaluated at a constant temperature (60 °C, Figure 4a), where the initial rates increased with the pressure until it reached a maximum value of 1.95 ± 0.09 mmol/min at 60 bar, while a further increment of pressure resulted in lower values of initial rates (0.70 ± 0.06 mmol/min at 80 bar). This finding is important from an economic and operational perspective, since the application of high pressure will increase the operational costs and be difficult to operate. Influence of temperature on the initial rates of lactose conversion was evaluated at a constant pressure (60 bar, Figure 4b). The highest values of initial rates were obtained at 60 °C, and further elevation of the temperature did not significantly increase the initial rates. Overall, the magnitude of the initial rates was higher by increasing the temperature than by the increment of the pressure. This observation is in agreement with the Arrhenius and Eyring theory [23].

### 3.3. Conversion and Yield

**Figure 5:**
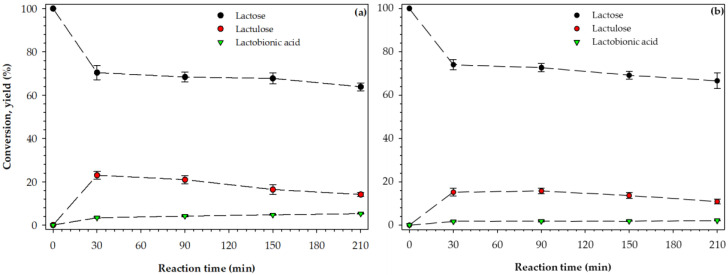
Conversion of lactose and yield of lactulose and lactobionic acid over time in: (**a**) sweet whey permeate (SWP), and (**b**) acid whey permeate (AWP). Temperature = 60 °C and pressure = 60 bar, and stirring rate 600 rpm.

Figure 5 shows the kinetic curves of lactose conversion for SWP and AWP. During the first 30 min of reaction, about 30 and 24% of lactose was converted for SWP and AWP, respectively. After 210 min, the conversion of lactose reached a maximum value of about 37 and 34% for SWP and AWP, respectively. Mäki-Arvela et al. [24] reported conversion values of about 90% during the oxidation of LA (0.86 M) under alkaline conditions (pH = 8), using Pd/C as a catalyst. The difference in the conversion values can be explained by the deactivation of the catalyst and the pH of the solution. Indeed, Mäki-Arvela et al. [25] reported that Ru/C rapidly underwent deactivation during the oxidation of lactose monohydrate solution (0.86 M), achieving conversion values of 28–32%. In SWP and AWP, the presence of minerals and residual proteins seems to impact the activity of the catalyst negatively. However, the deactivation of Ru/C due to minerals and residual protein needs further experimental evidence.

Figure 5a,b showed the formation curves for LAU and LBA corresponding to SWP and AWP, respectively. In untreated samples of SWP and AWP, the concentration of LAU and LBA was below the detection limit. An increment in the yield values of LAU was observed within the first 30 min of reaction, 22.99 ± 0.81 and 17.29 ± 0.96% for SWP and AWP, respectively. The relatively high yield values for SWP is not surprising, since the pH of the SWP (6.23 ± 0.01) favored the formation of LAU. Seo et al. [26] reported yield values of LAU of 29% (90 °C and 20 min of reaction) during the isomerization of cheese whey, using Na_2_CO_3_ (0.5%) as a catalyst. As the reaction proceeded, the yield values decreased to 14.13 ± 0.13 and 10.93 ± 0.07% for SWP and AWP, respectively. The observed reduction in the yield of LAU by increasing the reaction time is due to the hydrolysis of LAU and subsequent formation of organic acids. The mechanism of lactose isomerization consisted of epimerization and aldose–ketose interconversion. Such a network of reactions is known as Lobry de Bruyn-van Ekenstein transformation. Hajek et al. [27] have studied the isomerization of lactose in an alkaline environment.

In the case of LBA, the yield for the SWP (Figure 5a) increased asymptotically with the reaction time, reaching a value of 5.23 ± 0.02%. Similar behavior, but less pronounced, was observed in the yield values of LBA for AWP (2.15 ± 0.15%). The relatively high yield of LBA for SWP, nearly 2-fold than AWP, is not surprising, since the pH of SWP (6.23 ± 0.01, Table 1) favored LBA formation. It is thought that acidic conditions hinder the interaction between molecular oxygen and LA moieties, affecting LBA formation. The role of pH during the catalytic oxidation of LA has been discussed elsewhere [28,29]. Mechanistically, the oxidation of aldehydes is consensually thought to occur in two steps, hydration of the aldehyde group and subsequent dehydrogenation to produce their corresponding acid [30].

### 3.4. Formation of Glucose, Galactose, and Organic Acids

**Figure 6:**
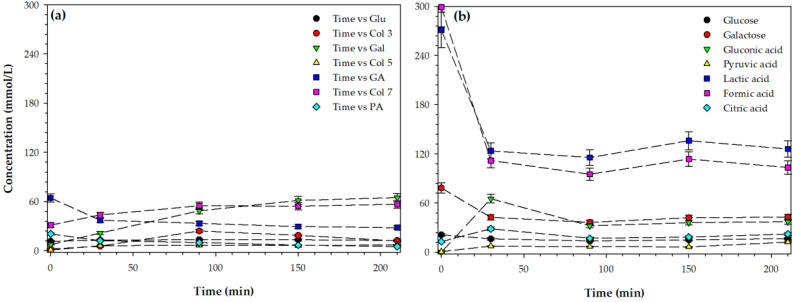
Formation of organic acids over time in: (**a**) sweet whey permeate (SWP), and (**b**) acid whey permeate (AWP). Temperature = 60 °C and pressure = 60 bar, and stirring rate 600 rpm.

The conversion values of lactose, as well as the yield values of LAU and LBA (Figure 5), suggest the formation of other products derived from hydrolysis, oxidation, and degradation. These products were divided into three groups—monosaccharides, sugar acids, and organic acids (Figure 6). The first group of compounds is monosaccharides (glucose and galactose) that are formed due to the hydrolysis of lactose, breakage of the glycosidic linkage. The initial concentration of glucose and galactose was higher in AWP (about 21 and 78 mmol/L, respectively) than that of SWP (about 4.61 and 2.21 mmol/L, respectively). Acid whey permeate is the byproduct of yogurt manufacture [31], and it is characterized by a relatively high concentration of organic acids, Table 1. As the reaction proceeded, the glucose in SWP (Figure 6a) gradually increased with time, reaching a maximum of about 13 mmol/L after 90 min. Subsequent progression of the reaction resulted in a lower concentration of glucose (about 9 mmol/L after 210 min). A similar trend was observed for galactose in SWP (Figure 6a), where a maximum concentration (24 mmol/L) was obtained after 90 min, followed by a reduction in the concentration with the progression of the reaction. On the other hand, the concentration of glucose and galactose in AWP (Figure 6b) gradually decreased with time from about 21 and 78 mmol/L at the beginning of the reaction to 16 and 42 mmol/L after 210 min, for glucose and galactose, respectively. It appears that lactose underwent hydrolysis to produce glucose and galactose that further oxidized to form their respective sugar acids, gluconic and galactonic acid.

The sugar acids represent the second group of components derived from the catalytic conversion of lactose. In SWP, the concentration of gluconic acid increased with time until it reached a plateau at 150 min (60 mmol/L). Afterward, the concentration of gluconic acid marginally increased up to 66 mmol/L after 210 min. In the case of AWP, the concentration of gluconic acid reached a maximum value of 65 mmol/L only after 30 min, followed by a reduction to about 30 mmol/L. Chemical process for the production of gluconic acid consisted of catalytic oxidation of a concentrated glucose solution under alkaline conditions [32].

The third group of components under consideration corresponds to organic acids. Overall, the concentration of organic acids in AWP was higher than that of SWP. Formic and lactic were the most predominant organic acids (299 and 270 mmol/L, respectively) in AWP. This observation is not surprising, since acid whey is derived from the formation of lactic acid bacteria, whose main product is lactic acid. A general trend was observed in SWP, where the concentration of organic acids increased slightly with the reaction time (Figure 6a). Citric, acetic, and uric acids were found at relatively low concentrations. These findings are in agreement with the earlier studies on the formation of isosaccharinic acids and organic acids [26]. Organic acids are formed from the breakdown of glucose and galactose. More specifically, lactic acid is formed as the primary byproduct of the catalytic oxidation of glucose [33]. The catalytic oxidation of glucose over bimetallic catalysts has been recognized as an efficient alternative for the production of gluconic acid. The oxidation of glucose occurs via a carbonyl conjugated radical mechanism due to the formation of H_2_O_2_, which in turns is formed by the presence of oxygen in the aqueous media [34]. However, the mechanisms of glucose oxidation strongly depend on the pH of the medium and the type of catalysts [33]. On the other hand, the concentration of organic acids in AWP showed an increase with time for citric and pyruvic acid, while the concentration of formic and lactic acid decreased with time (Figure 6b). Citric acid and pyruvic acid are formed due to glucose breakdown, which subsequently formed oxalic acid and others.

### 3.5. Product Distribution

**Figure 7:**
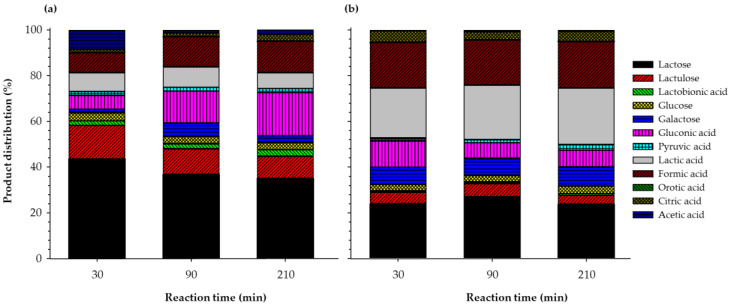
One-pot synthesis of lactose derivatives from (**a**) sweet whey permeate and (**b**) acid whey permeate.

Figure 7 exemplifies the one-pot synthesis of lactose derivatives, where a pool of molecules synthesized from SWP (Figure 7a) and AWP (Figure 7b) was plotted at different reaction times. In SWP, the more predominant compounds were lactose (43–35%), gluconic acid (6–18%), LAU (14–9%), formic acid (8–13%), and lactic acid (8–7%), whose concentration varied with the reaction time. For AWP, lactose (27–23%), lactic acid (21–24%), formic acid (19–20%), gluconic acid (11–7%), and LAU (5–3%) were the most predominant compounds. It is worth mentioning that these results should be considered cautiously because the final concentration is influenced by a number of factors, including pH, temperature, the composition of the stream, the type of catalyst, and catalyst load. Thus, the concentration of a given group of components may be optimized in order to maximize their production according to the study of reaction kinetics. The concept of one-pot was first introduced by Kolb et al. [35], who described a click-chemistry in which a set of chemical transformations yielded higher efficiency, fast rates, and simple product isolation. Soon after, the concept of click chemistry quickly found widespread application in various research areas, including organic chemistry, polymer synthesis, petroleum, and biorefinery. Currently, there are several terminologies to describe multi-step reactions that take place in one pot, such as domino reaction, cascade reaction and tandem reaction. One-pot synthesis is effective because several chemical transformation steps can be carried out in a single pot, while circumventing several purification procedures at the same time. Thus, a one-pot procedure can minimize chemical waste, save time, and simplify practical aspects.

## 4. Conclusions

The one-pot conversion of lactose streams resulted in the formation of four main group of components—rare carbohydrate (lactulose), monosaccharides (glucose and galactose), sugar acids (gluconic acid), and organic acids. The final distribution and their concentration differ from SWP and AWP, where lactulose was favored in SWP, and organic acids were favored in AWP. The synthesis of a pool of molecules through a one-pot approach represents an alternative approach for the utilization of streams of lactose. Upon further separation, organic acids can be used as building blocks of numerous applications in the manufacture of herbicides, bioplastics, and biofertilizers.

## Figures and Tables

**Table 1:** Physicochemical characteristics of sweet whey permeate and acid whey permeate.

Parameters	Sweet Whey Permeate	Acid Whey Permeate
pH	6.23 ± 0.01	4.38 ± 0.02
Total solids (g/L)	87.38 ± 0.25	85.35 ± 0.06
Total non-volatile solids (g/L)	8.23 ± 0.17	9.22 ± 0.16
Fat (g/L)	0.85 ± 0.23	1.81 ± 0.10
Total protein (g/L)	3.02 ± 0.01	3.95 ± 0.08
Lactose (g/L)	76.42 ± 4.3	61.73 ± 6.29
Organic acids (g/L)	7.11 ± 0.35	17.91 ± 0.89

Mean ± standard deviation (*n* = 3).
